# Dimpled SiO_2_@γ-Fe_2_O_3_ nanocomposites – fabrication and use for arsenic adsorption in aqueous medium†

**DOI:** 10.1039/d0ra09907d

**Published:** 2021-01-05

**Authors:** Saruta Deeprasert, Lilin Wang, Konstantinos Simeonidis, Nguyen Thi Kim Thanh, Etienne Duguet, Stefanos Mourdikoudis

**Affiliations:** Biophysics Group, Department of Physics and Astronomy, University College London London WC1E 6BT UK s.mourdikoudis@ucl.ac.uk ntk.thanh@ucl.ac.uk; UCL Healthcare Biomagnetic and Nanomaterials Laboratories 21 Albemarle Street London W1S 4BS UK; Department of Physics, Aristotle University of Thessaloniki 54124 Thessaloniki Greece; Univ. Bordeaux, CNRS, Bordeaux INP, ICMCB, UMR 5026 F-33600 Pessac France etienne.duguet@icmcb.cnrs.fr

## Abstract

We report the synthesis of nanocomposites made of silica nanoparticles whose six surface dimples are decorated with magnetic maghemite nanoparticles and their use for detection and recovery of arsenic in aqueous media. Precursor silica nanoparticles have aminated polystyrene chains at the bottom of their dimples and the maghemite nanoparticles are surface functionalized with carboxylic acid groups in two steps: amination with 3-aminopropyltrimethoxysilane, then derivatization with succinic anhydride in the presence of triethylamine. In the end, the colloidal assembly consists of the regioselective grafting of the carboxylic acid-modified iron oxide nanoparticles onto the 6-dimple silica nanoparticles. Several characterization techniques such as transmission electron microscopy (TEM), Fourier-transform infrared spectroscopy (FTIR), dynamic light scattering (DLS) are employed to assess the grafting process and study the influence of the maghemite functional groups on the quality of the composites formed. The resulting magnetic nanocomposites are used for the environmentally benign detection and removal of arsenic from aqueous medium, being readily extracted through means of magnetic separation.

## Abstract 2

The process of grafting maghemite NFs onto silica dimples.

## Graphical abstract

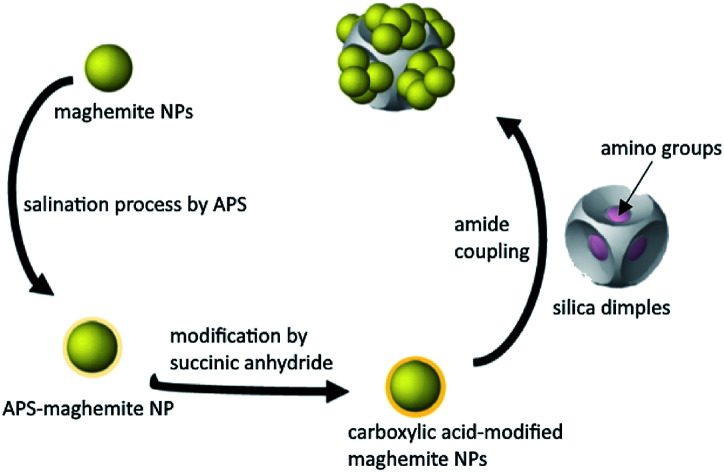

## Introduction

**Scheme 1:**
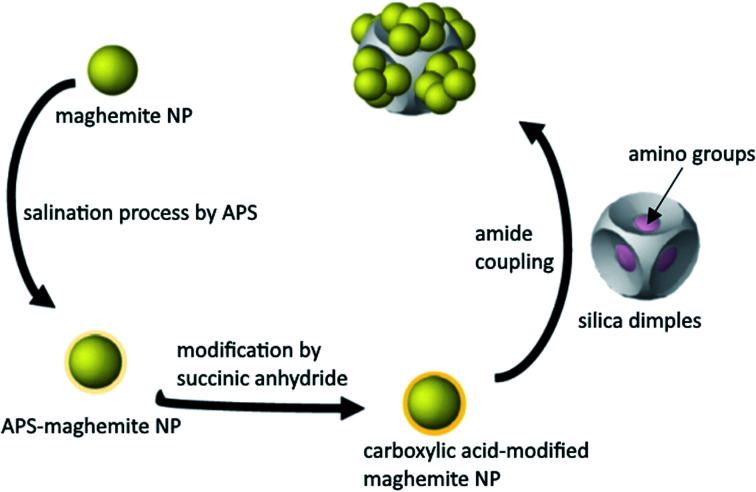
The process of grafting iron oxide nanoparticles onto the silica dimples.

Even though there are several synthetic pathways to produce different types of nanocomposites, it is still challenging to precisely control their composition, shape and yield. In a given nanocomposite composed of a magnetic component and another part, the former component endows desirable magnetic properties (such as ability for magnetic separation) whereas the second component will offer different functionalities, such as catalytic ones.^1^ The shape of a nanocomposite is another particular feature that draws attention due to its crucial role for the resulting physical properties. Core–shell, core–satellite, dimer or oligomer heterostructure configurations are some of the frequently observed morphologies in different types of nanocomposites.^2^ Hybrid nanostructures that combine different materials in a sole nanostructure, such as metal–metal, metal–metal oxide, metal–metal chalcogenide and metal–dielectric can present the properties of both types of materials, with the possibility of the appearance of new, enhanced, or synergistically improved properties.^3–5^ Regarding the yield, it is important to produce nanocomposites in large scale aiming for real-world applications, with high product homogeneity, monodispersity and quality. Purifying methods such as magnetic separation and centrifugation do not always ensure an easy removal of any by-products, therefore it is better to aim for a production method that is efficient and sufficient.^6^ Various synthetic routes have been suggested to generate nanoarchitectures with complicated shapes, including seed-mediated growth or self-assembly of pre-synthesized colloidal structures.^7^ Our group has published the synthesis of dimpled symmetrical silica particles with a varying number and depth of dimples and managed to grow gold nanoparticles (NPs) onto these dimples.^8^ To achieve this, certain chemical modification steps were required in order to favour the chemical interaction between the SiO_2_ dimples and the Au NPs. Regarding magnetic nanocomposites, it has been shown that the growth or deposition of the magnetic part onto a non-magnetic material can be tricky when a large lattice mismatch is present between two crystalline components.^2^ When silica is a component of magnetic nanocomposites, it is mostly used as a shell to protect the magnetic core particles from aggregation, their chemical stability and reduce their toxicity. The use of a common Si source, tetraethyl orthosilicate (TEOS), in a sol–gel approach is a typical method to produce SiO_2_-coated magnetic nanocomposites.^9^ In the present work, we attempted to use our dimpled silica NPs as host particles, and decorate their exterior surface with ‘guest’ magnetic components, that is, iron oxide NPs. The amorphous structure of silica overcomes the aforementioned lattice mismatch issue, which would occur if both host and guest materials were crystalline. We thought that selective surface functionalization with suitable chemical groups would be a promising way to deposit magnetic NPs onto these non-magnetic components. In this way, the formation of this composite would be achieved even though there is no direct contact between the two parts, silica and maghemite: the reason is the presence of the aminated polystyrene chains at the silica dimples and the organic molecules at the maghemite part. Therefore, with this motivation in mind, we investigated the conditions that enable the assembly of maghemite NPs onto monodisperse dimpled silica NPs to produce SiO_2_/γ-Fe_2_O_3_ magnetic nanocomposites. We studied mostly the case of silica particles with 6 dimples, but the same process could be used for samples with different numbers of dimples. We also investigated the application of the resulting composites as magnetically separable adsorbents for the removal of arsenic from polluted water, which constitutes a promising approach for water purification technology. One of the biggest concerns upon the use of single magnetic NPs in such applications is that they may be prone to aggregation, mostly due to magnetic coupling, when subjected to a magnetic gradient. This can compromise their efficiency but can also complicate their fate after use. Therefore we seeked to find a way to increase the interparticle distance of the magnetic iron oxide NPs, through the formation of hybrid composites with enhanced properties and performance by grafting maghemite NPs on SiO_2_ dimples. This seemed a promising way to tackle the aforementioned aggregation risk. In this way, we aimed to increase the available surface area of the product and activate a higher number of sites for the attached particles (Scheme 1).

## Results and discussion

We have already shown that the morphology of the silica dimples can be controlled with high precision. Moreover, dissolving the initially grafted particles at the dimples would allow to regrow or decorate them with other types of particles upon desire. This grafting can be in principle achieved by forming an amide bond between the ‘guest’ NPs and the ‘host’ SiO_2_ dimples modified by a carboxylic acid group and an amino group, correspondingly.^6^

### Functionalisation of iron oxide nanoparticles

**Fig. 1:**
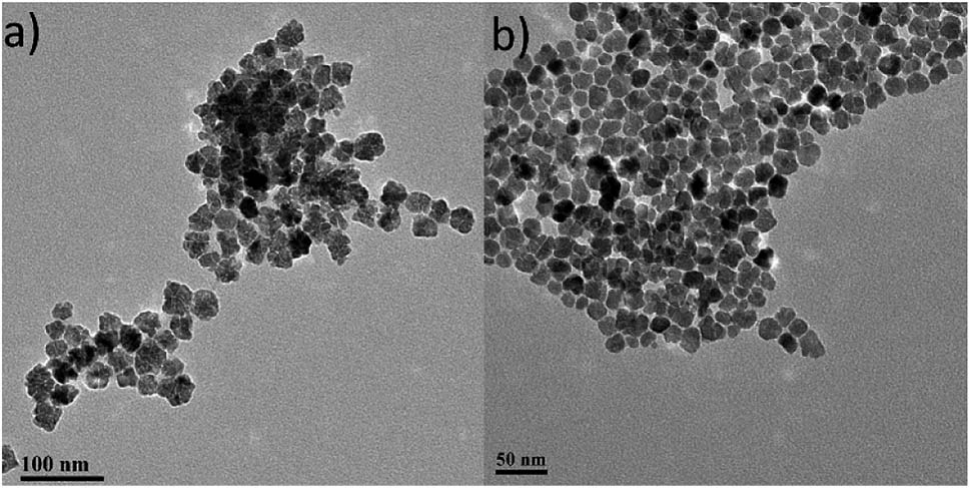
TEM images of uncapped (a) and citrate-capped maghemite NPs (b).

Iron oxide NPs, one of the most popular NPs systems with uses in a large variety of applications, are commonly studied in the environmental field for arsenic removal.^10^ There are many forms of iron oxide NPs that can be present depending on the preparation process and the desired role, where the most frequent ones are magnetite (Fe_3_O_4_) and maghemite (γ-Fe_2_O_3_). These iron oxide NPs are superparamagnetic in a certain size regime, they have low toxicity and are biocompatible; therefore they are suitable for environmental applications.^11^ Furthermore, with appropriate surfactants, iron oxide NPs can become stable in aqueous medium and can be used to remove several pollutants in drinking water, groundwater or wastewater.^10,12^ Before any surface modification of the iron oxide NPs used in our work, we characterized the as-prepared particles in order to determine fundamental properties such as colloidal stability and concentration, particle size, size distribution and surface functionality using standard techniques. In particular, to determine the particle concentration used in this study, a calibration curve was constructed *via* UV-Vis through obtaining spectra of samples of known iron concentration (Fig. S1†). Based on that curve, the iron oxide colloidal dispersion used in the current study had concentration values of 3.37 mg mL^−1^ (0.44 for the citrate-capped NPs – these polyol-route prepared iron oxide NPs can sometimes be functionalized with citrate anions in order to improve their colloidal stability at biological pH). The TEM images for both of the above samples are shown in Fig. 1.

**Fig. 2:**
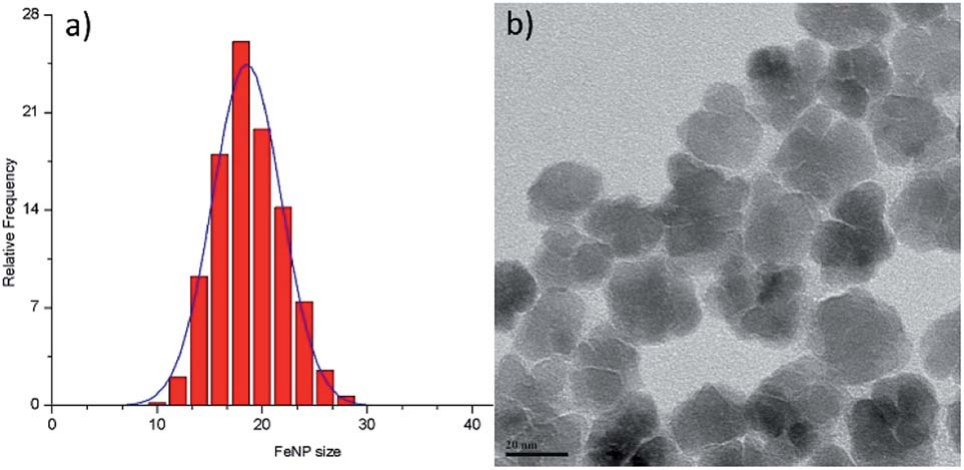
Size distribution histogram (a) and high-magnification TEM image (b) for the maghemite nanoflowers (NFs) studied.

Both ‘uncapped’ and citrate-capped γ-Fe_2_O_3_ NPs had an average TEM size of 18.9 ± 3.5 nm. The corresponding size distribution histogram is presented at Fig. 2a. In fact, DLS measurements show that the citrate-capped particles have an average size of about 32 nm, with an approximately 100 nm DLS size derived for the uncapped NPs (Fig. S2†).

**Fig. 3:**
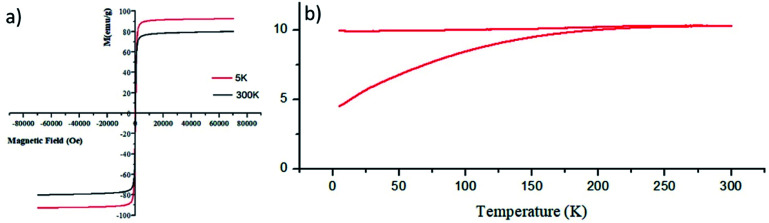
*M*–*H* plots at 5 K and at room temperature (a) as well as ZFC-FC measurements (b) for γ-Fe_2_O_3_ NFs.

**Fig. 4:**
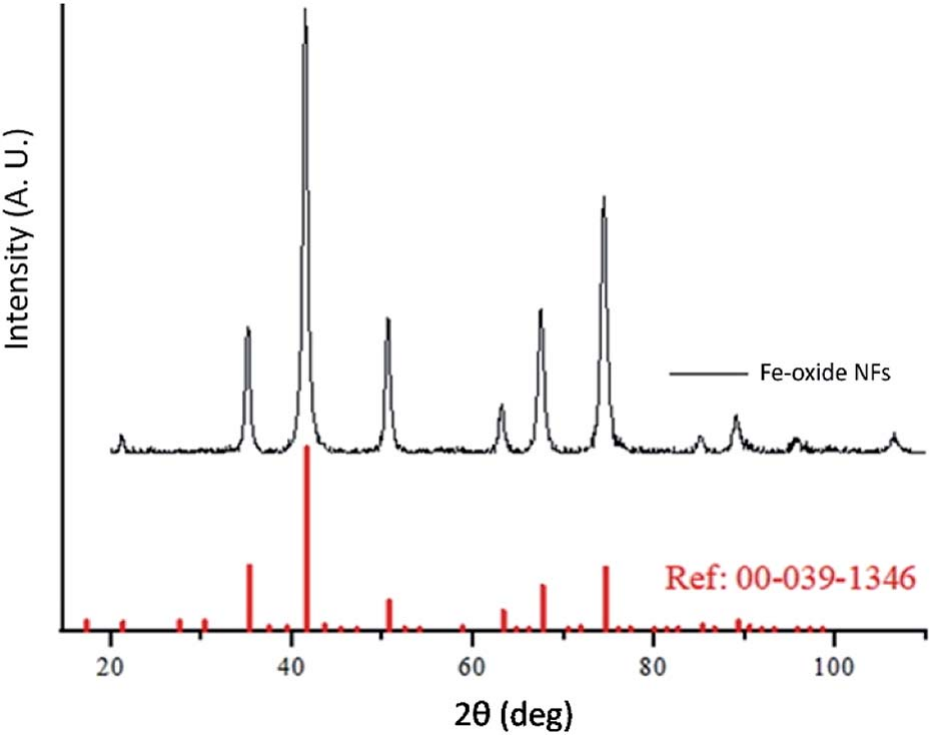
XRD measurement for maghemite NFs and corresponding peak positions of the relevant JCPDS pattern for γ-Fe_2_O_3_.

It has to be noted that even the particles with the biggest size are nearly superparamagnetic, as they possess a multi-core flower shape, with the diameter of each petal being of around 12 nm (see Fig. 2b). *M*–*H* plots reveal a coercivity close to 0 at room temperature, while ZFC-FC recordings show that the blocking temperature of these NFs is below room temperature (Fig. 3). The magnetization is normalized to the weight of the magnetic fraction: thermogravimetric analysis (TGA) measurements (Fig. S3†) have helped to identify the organic content, which is not magnetic. The saturation magnetization at room temperature coincides within uncertainty with the magnetic saturation of bulk maghemite (78 emu g^−1^).^13^ XRD measurements illustrate that the NFs are composed of maghemite, as the observed peaks match the expected ones from the JCPDS pattern for maghemite (00-039-1346), see Fig. 4 and Experimental section below. On the other hand, single-crystalline magnetite particles of a size over 30 nm would exhibit clear ferromagnetic behaviour.^14^ Overall, the particles were relatively homogeneous in size and shape with a slight tendency for aggregation, mostly occurring for the uncapped NPs, as shown also by the larger DLS size. Apart from the surface chemistry, the size of iron oxide NPs plays a significant role in a range of physical properties, not limited to the magnetic behaviour.^15^ Therefore, we compared the efficiencies of these two types of iron oxide NPs in the silica dimples decoration quality.

To confirm the presence of NPs functionalization before moving on with the further experimental steps, the pre-synthesized citrate-capped maghemite particles were studied by FTIR measurements. At Fig. S4a† a spectrum of the initially synthesized iron oxide NPs is shown and the peaks at around 1397 cm^−1^, 1598 cm^−1^ and 3409 cm^−1^ indicate the existence of citrate on the capped iron oxide NPs. The latter peak is assigned to the –OH stretching of citrate.^16^ The *ζ*-potential of the particles was also measured, in order to evaluate the degree of the colloidal dispersion stability. Normally, for a highly stable dispersion, the *ζ*-potential should be around ± 30 mV or over.^17^ Furthermore, since the pH level can affect the *ζ*-potential value, its measurement in a range of different pH is typically used to identify the isoelectric point of the NPs.^16^ In our NPs, the isoelectric point was at around pH 6.5. From the Fig. S5,† it can be observed that in particularly acidic conditions (*e.g.* at pH 4), the NPs are highly stable with a *ζ*-potential of *ca.* ± 30 mV. Especially the pH level of the particle dispersion is a crucial parameter that needs to be adjusted carefully to ensure optimum conditions to induce the attachment of functional groups at the particle surface during the next stage, such as the peptisation step.^19^ Therefore we decided to evaluate also visually the dispersion behaviour of the iron oxide NPs at different pH values, which were adjusted by adding diluted nitric acid. Fig. S6† illustrates that the particles dispersion displayed a different colour as soon as some nitric acid was added, but the particles were relatively stable until approximately 1 mL nitric acid was introduced. By continuing this process of strengthening the acidic conditions, it was observed that precipitates started to form at the bottom of the vial (Fig. S6†). A higher degree of precipitation was noticed progressively. This behaviour can be explained by the fact that the pH of the solution can affect the solubility of any dispersed molecules. If the pH value induces the conditions where the dispersed molecules (*e.g.* capping agents) will possess no net electric charge, then the latter compounds will have very low solubility and can precipitate out of the solution.

In terms of colour changes, the colour of the as-produced dispersion was dark brown, but it got a lighter hue after acid addition. This has likely resulted from the changes in the stability and the magnetic properties or the shape structure of the NPs, as reported by Mufti and co-workers.^20^ Therefore, in that point it was clear that it is essential to spot any pH changes taking place and to constantly assess the particles stability, since precipitation is unfavourable. In our work, 3-amino-propyltrimethoxysilane (APS) was then coated onto the iron oxide NPs followed by a carboxylic acid group, succinic anhydride, *via* peptidic bonding (known also as amide coupling), aiming to produce a properly functionalized material that will be grafted onto the amine-functionalised silica dimples. We have already shown that those chemical reagents have been successfully used for the grafting process of other materials onto SiO_2_ dimples, thus, we decided to adopt this process for the current system.^19^ Among other coupling agents available, APS and succinic anhydride demonstrated the highest efficiency for surface modification.^21,22^

The silanation process did not affect the morphology of our iron oxide NPs. As shown at the TEM images of Fig. S7,† the APS modification of the particles surface did not affect the size or shape of the particles. Still, a minor degree of aggregation was noticed, which indicates that the APS alone cannot completely prevent any cluster formation of the NPs. Thus, if the iron oxide NPs are unsupported (if they are not combined with ‘host’ foreign particles), their colloidal stability, overall magnetic properties and performance in applications could be perturbed because of some aggregation tendency.^23^ Of course, APS is not a voluminous molecule that could create a visible contrast at the TEM, therefore, we carried out additional characterization at this stage. After doing *ζ*-potential measurements in a range of pH values, the isoelectric point of the APS-functionalized particles was determined at about 10.5 (Fig. S8†). After a heat treatment at 110 °C, the isoelectric point of the NPs turned to 10 whereas according to the literature, the isoelectric point of the major charge carrier of the primary amine is 10.5.^19^ As the silanation process occurs between APS and the iron oxide NPs, the above result shows that the heat treatment enhances the oxidation reaction of the present alkoxysilane on the APS which then improves the modification efficiency. Furthermore, it also implied that there is a high density of APS molecules attached on the surface of the NPs.^19^ The significant increase of the isoelectric point of the particles after the modification with APS is a strong indication of their successful surface functionalization. In fact, aiming to find the optimum silanation reaction conditions, we tested different dehydration and degassing processes, such as using vacuum–N_2_ inert gas Schlenk line techniques and/or a rotary evaporator, but the results were pretty similar in all cases.

To further confirm the particle modification, FTIR was also employed to characterize the NPs at this stage. The comparison of the peaks of a standard APS sample and of our APS-modified iron oxide NPs indicated the presence of APS on the NP surfaces. Spectra which were similar in a good extent were observed (Fig. S4b†), with peaks at 805, 1090, 1265 and 2923 cm^−1^ for the modified NPs. In particular, the peak at 2923 cm^−1^ can be assigned to the bending vibrations of C–H.^16^ Table S1† contains a list of the standard APS peaks. Moreover, by comparing the FTIR spectra of Fig. S4b† with those of Fig. S4a,† the changes in the peaks can be clearly spotted. More specifically, the peaks at 1397 and 1598 cm^−1^ can barely be observed anymore while the aforementioned peaks at 805, 1090, 1265 and 2923 cm^−1^ were new. Thus, this result corroborates the *ζ*-potential (isoelectric point) results regarding the successful coating of APS onto the iron oxide NPs.

Further modification of the APS-functionalised iron oxide NPs was carried out so as to provide the carboxylic acid group on their surface. This step was required before grafting the particles onto the amine-functionalized silica dimples. Therefore, the amino group on the exterior layer of the NPs surface was converted to carboxylic acid group. Fig. S9† shows that the isoelectric point of the carboxylic acid-modified maghemite NPs decreased from roughly 6.5 to 4.5 after heat treatment. The observed value for the p*K*_α_ value is consistent with the p*K*_α_ of conventional carboxylic acids. In case of any remaining amino groups on the surface of the iron oxide NPs, they can be converted to carboxylic acid moieties by adding more triethylamine (TEA) into the sample. It is noticed that the thermal treatment lowers down the isoelectric point of the carboxylic acid-modified NPs, even though there is already a dramatic drop of its value once succinic anhydride was added. This indicated that the heat treatment had successfully removed ethanol, a by-product which can appear in the obtained modified NPs, but it also helped to increase the carboxylic acid load on the NPs surface. Similarly, also in this case, different degassing-dehydration approaches were tested using Schlenk line and/or rotary evaporator, with a reproducible value for the isoelectric point of the obtained NPs.

At this stage, the NPs underwent also a FTIR characterization as shown at Fig. S10.† According to previous research,^21^ the modification of gold NPs with succinic anhydride would lead to the appearance of peaks at about 1650 and 1730 cm^−1^, demonstrating the presence of carboxylic acid.^16^ In our case, peaks with somewhat shifted positions from the expected ones were noticed, at 1676 and 1767 cm^−1^, respectively. These peaks can be assigned to the C=O amide stretching and C=O stretching of the carboxylic acid. A small peak at around 2929 cm^−1^ can be assigned to C–H stretching. In addition, by comparing this FTIR spectrum to the one concerning the APS-modified particles (Fig. S4b†), stronger and additional peaks can be clearly seen. In particular, peaks at higher frequencies can be barely noticed in the case of carboxylic acid-modified NPs (Fig. S10†). This is a strong indication of the attachment of the acid entity on the surface of the NPs.

### Iron oxide NPs grafting onto silica dimples

The silica dimples are introduced to enhance the performance of the NPs by grafting the maghemite particles onto the dimples and generating a kind of ‘hybrid’ composite material. The carboxylic acid-modified iron oxide NPs were decorated onto the SiO_2_ dimples *via* peptidic bonding. We used six-dimpled silica NPs as host material, whereas either citrate-capped or initially uncapped iron oxide NPs were used as guest NPs. Of course, all modifications (APS, succinic anhydride, *etc.*) were carried out in both ‘uncapped’ and citrate-capped γ-Fe_2_O_3_ NPs. These different combinations were investigated aiming to find the optimum conditions for the decoration of iron oxide NPs onto the dimples.

Firstly, the combination between the dimples and the citrate-capped maghemite NPs was tested. A rotary evaporator was employed in order to control efficiently the dehydration stage as the heat treatment plays an important role in the grafting process.

**Fig. 5:**
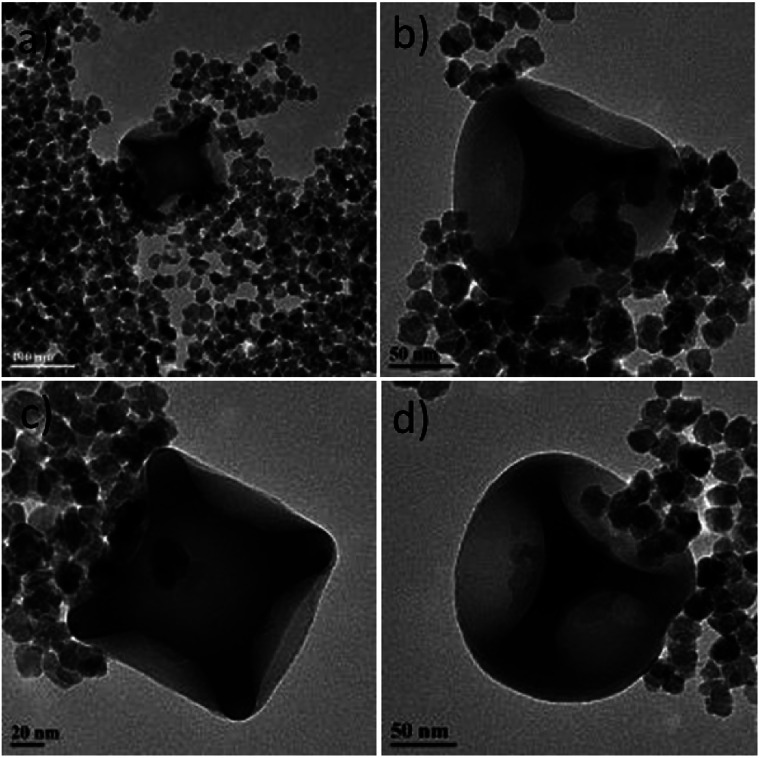
TEM images of carboxylic acid-modified citrate-capped iron oxide NPs grafted on six-dimpled silica NPs. A colloidal dispersion of this sample is depicted at Fig. S11a.† The images correspond to different regions of the TEM grid studied.

Fig. 5 presents the TEM images of the sample under discussion and it seems that there is only a partial tendency for the attachment of the iron oxide NPs onto the silica dimples. A kind of lateral attachment is present, but probably the amide coupling between the silica host particles and the iron oxide guest NPs was only partly successful. At the same time, a good amount of free (not bound) iron oxide NPs can be also spotted, which is rather expected due to the fact that excess concentration of those particles was used in comparison to that of the silica dimples. This was done deliberately in order to ‘force’ statistically more ‘collisions’ between the former particles to the dimples.

**Fig. 6:**
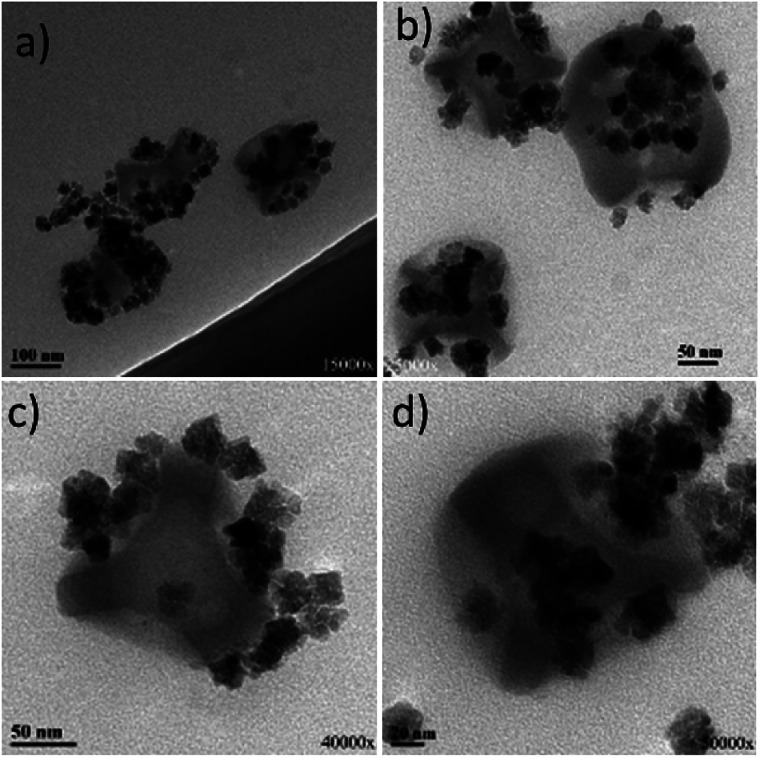
TEM images of carboxylic acid-modified uncapped iron oxide NPs grafted on six-dimpled silica NPs. A colloidal dispersion of this sample is depicted at Fig. S11b.† The images correspond to different regions of the TEM grid studied.

The combination of initially ‘uncapped’ iron oxide NPs with silica dimples was also examined. Fig. 6 illustrates the TEM images of those carboxylic acid-modified NPs grafted onto the dimples. In comparison to Fig. 5, it can be noticed that there is a quite visible difference in the attachment behaviour between the iron oxide particles and the silica structures. The maghemite particles were successfully attached to a big majority of the dimples present in the reaction pot. Moreover, the apparently nice arrangement of the iron oxide NPs confirmed that the NPs did not randomly ‘float’ next to the dimples, but formed an amide bonding with the dimples, demonstrating an attachment behaviour not only in a lateral mode, but also in a kind of ‘main body decoration-type attachment’. This is because it is unlikely for the maghemite NPs to align perfectly around the silica nanostructures without having any interparticle interactions. It has to be noted that a small portion of free iron oxide NPs is still present, since an excess of them was used in the beginning of the grafting process, aiming to enhance the possibilities of dimple decoration. These unbound particles were present even after repeating washing procedures *via* centrifugation. Ideally, a slightly lower concentration of iron oxide particles could be possibly adjusted in order to reduce the number of unbound particles. However, it is important to note that almost no ‘free’ silica dimples were spotted during TEM observation, denoting a rather successful grafting process.

Based on the above observations, it seems that the different surface functionalization stages may have been easier to fully accomplish in the ‘uncapped’ iron oxide NPs, compared to those capped with citrate. In that way, the amide bonding could be more efficient for the former type of particles, leading to a better grafting ability. Furthermore, there is some possibility that the size of the γ-Fe_2_O_3_ NPs affects their grafting performance as the DLS size of the as-prepared citrate-capped iron oxide NPs is smaller than that of the uncapped ones. Possibly, particles of a larger size could attach more effectively onto the dimples, by sharing a bigger interface with them. But this could be regarded as only a secondary potential reason with lower importance, since, for example, gold NPs of an even smaller size (12 nm) have been successfully grown onto silica dimples.^8^

Our present results show that in principle our strategy to decorate silica dimples with foreign particles can be extended from noble metals like gold to other systems such as iron oxides. In our previous works, when we managed to combine gold NPs and silica structures in a range of configurations (not necessarily with gold being always located at the outer layer of silica), in most cases, pre-synthesized gold NPs were used.^7,8,24–27^ However, we also managed to obtain a regrowth stage for gold by using Au NP seeds, in somewhat different approaches.^6,28^

Nanocomposites containing plasmonic satellite elements, such as gold, can find applications as photonic materials, superlenses and sensors or in life sciences, optics and coating industry.^7^ The nanocomposites of the present work were employed for As removal in water as described below.

### Arsenic removal

**Fig. 7:**
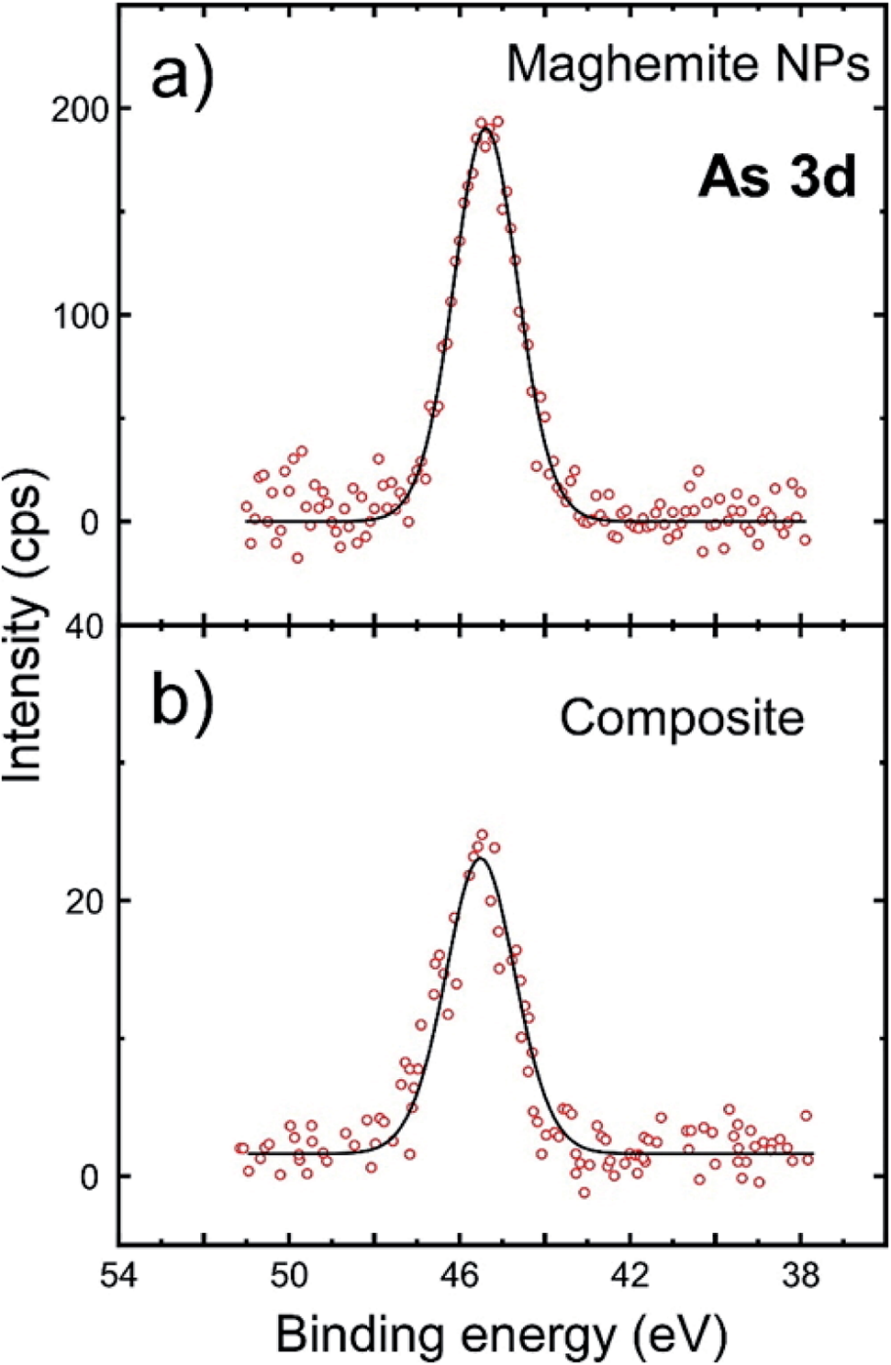
The sorption of As(v) studied by high-resolution XPS spectra at the As 3d: (a) iron oxide NPs, (b) silica dimples@iron oxide NPs (hybrid composite of Fig. 6).

The combination of magnetic components and proper supports for water purification was pursued, since it allows the magnetic separation of the used material together with the adsorbed pollutants, which can be organic contaminants, microorganisms and heavy metals. For instance, pine-derived biochar has been employed as a support to stabilize Fe(0) NPs to remove As(v). The biochar-supported-nanoscale zerovalent iron NP composites were produced by precipitation the NPs on carbon surfaces. Batch, continuous flow and completely mixed reactors were examined to assess the efficiency of the As removal. Surface complexation seemed to be the principal removal mechanism, though a reduction process might also have taken place.^29^ Silica has also been combined with magnetic NPs, usually Fe_3_O_4_ ones for water purification. In these cases, silica will usually act as a coating material of the core magnetic particles, protecting them from corrosion, while not perturbing their magnetic properties. For example, sulfur-doped Fe_3_O_4_@SiO_2_–amide-linked organic polymer was produced by chemically anchoring the organic polymer onto the amine-modified magnetic NPs. These composites showed high adsorption selectivity for Hg(ii) uptake from aqueous medium.^30^ In another work, Fe_3_O_4_@silica–xanthan gum composites were fabricated by fixing the natural polymer xanthan gum on the surface of magnetite microspheres *via* a sol–gel method. Those composites were applied to remove Pb(ii) from water. Rapid adsorption with prolonged recyclability was achieved.^31^ In our case, as shown above, the silica dimples act as the ‘host’ of the iron oxide NPs, aiming to ensure that they are kept relatively ‘isolated’ with sufficient interparticle distances, in order to prevent their aggregation. In addition, the heavy dimples are thought to facilitate the control of the fate of the magnetic particles after their application, not only through means of magnetic separation, but also *via* centrifugation, if required. Such stability has the potential to feature these systems with high repetition response for the detection of low pollutant concentrations whereas the presence of silicon oxide appears advantageous towards implementation for sensor devices. The maghemite NPs, if kept ‘decorated’ onto the surface of the dimples, and not ‘aggregated’, they retain a relatively high total surface area which may be of value in a range of applications, including sensing. In this regard, we examined the efficiency of our nanocomposites, especially of the sample shown at Fig. 6 as adsorbing agent in a timely topic, the remediation of water contaminants and the selective capture of toxic pollutants. Arsenic is a quite toxic element responsible for a variety of health problems so far in mankind. Potential adsorbents should provide high affinity to aqueous arsenic species in the pH range 6–8. As(v) is the dominant speciation state in polluted groundwaters. The corresponding oxy-anion appears negatively charged H_2_AsO_4_^−^/HAsO_4_^2−^. Hence, we decided to carry out certain tests to evaluate the efficiency of our composites to detect and capture arsenic from aqueous medium. We examined the relevant activity of silica dimples@maghemite NPs (depicted in Fig. 6) as well as of unsupported maghemite NPs in water spiked with As(v). In both cases, no modification of As(v) oxidation state was observed, as shown in Fig. 7. The As(v) spectrum contains a sole peak at 45.3 eV which indicates the existence of As(v) species.^32^ Binding of As(v) oxy-anions takes place by the formation of inner-sphere complex between surface Fe^3+^ and As(v) through oxygen bridges. Prior hydrolysis of surface maghemite is a very possible scenario with the potential to enhance affinity of As(v) species.^33^

The As-to-Fe mass ratio was derived through a quantitative analysis of XPS spectra. XPS is a sensitive technique which can accurately determine the quantity of As at the surface of materials even when no detectable variation of its concentration in the liquid phase could be measured by the most sensitive atomic absorption method. So, the As-to-Fe mass ratio regarding As(v) adsorption was higher for the sole maghemite NPs, reaching 0.10, with a somewhat lower performance of the hybrid iron oxide NP-dimple composite (0.05). The lower As extraction yield with the nanocomposite structure could be explained by the fact that a non-negligible part of the maghemite NPs is dedicated to the anchoring of the NPs at the bottom of the dimples. So the ‘active’ maghemite surface area is lower in the nanocomposite than in the free NPs. In addition, the functionalization steps for the surface of the grafted maghemite NPs might have slightly deteriorated their ability for As adsorption in comparison to the as-prepared ‘uncapped’ particles. Still, the composites' performance can still be regarded as competitive. This is because an important factor regarding the use of engineered nano-systems in water technology is the life cycle of used NPs. Consumed NPs should be carefully handled, aiming to prevent or minimize their leakage to the environment. In this context, the design of nanocomposites as the present hybrid composite, which allows an easy handling and removal through magnetic separation or even centrifugation means, is beneficial. In the current work, even if single γ-Fe_2_O_3_ NPs showed a better affinity towards As remediation, their small size and the possibility for random aggregation could complicate their safe disposal after the As adsorption or their detection response to very low arsenic concentrations, especially in a ‘real-world’ (and not laboratory) application scale.^10^ Still, considering that the hybrid composite displays lower efficiency than previous nanoscale materials on As removal, reported by us^32^ or others,^34^ the system presented herein could be suggested to act as a supplementary approach for water treatment, with the main objective being the sensing of the presence of potentially dangerous pollutants in aqueous medium such as water destined for drinking. On this concept, the studied system shows a proportional correlation with arsenic concentration as expressed by the measurement of As-to-Fe ratio for various initial arsenate concentrations in the range 1–20 mg L^−1^ (Table S2).†

High resolution XPS spectra at O 1s (Fig. S12†) indicate a peak at 530 eV being attributed to (Fe–O) whereas another one at 532.1 eV is assigned to surface hydroxyl groups. The peak at 533.9 eV corresponds to HAsO_4_^−^ and appears stronger for the sample composed of unattached maghemite NPs. Multiple peaks in the composite are also related to the massive presence of SiO_2_. Fig. S13† shows that very similar spectra were obtained at high resolution XPS spectra focused at Fe 2p. Peaks located at around 710.5 eV (Fe 2p_3/2_) and 724.2 eV (Fe 2p_1/2_) are analysed in two contributions indicating the presence of mixed oxidation states of iron. This means that the maghemite (γ-Fe_2_O_3_) NPs may contain a small portion of magnetite (Fe_3_O_4_) phase, too. Overall, the adsorption mechanism for arsenate species on the studied silica dimples@iron oxide NPs is suggested as follows: at pH 6, As(v) is met almost exclusively in the monovalent oxy-ionic form H_2_AsO_4_^−^ according to its known speciation. Such species requires one active site by the iron oxide surface to be absorbed, which is probably provided upon its approach to the nanoparticles surface, by non-occupied Fe^3+^ sites or after exchange with carboxylic acid molecules attached onto it. In the last case, the detachment of each carboxylic acid chain may release three adsorption sites. After that, H_2_AsO_4_^−^ is chemisorbed forming complexes with one Fe^3+^ ion from the solid phase (mononuclear) through monodentate or bidentate oxygen bridges. The effect of acidity was verified by the determination of As-to-Fe ratio for different pH values (Table S2†). As the adsorption pH increases to the alkaline region, the domination of the bivalent HAsO_4_^2−^ species which require two adsorption sites and the enhancement of negative surface charge appears to inhibit adsorption efficiency.

## Conclusions

In summary, iron oxide NPs, after successful surface modification stages, were decorated onto silica dimple structures to obtain magnetic nanocomposite NPs. The grafting of the maghemite NPs onto the dimples was achieved through amide bonding. A series of characterization techniques such as *ζ*-potential measurements and FTIR spectra were employed to monitor each separate reaction or functionalization step, to ensure that the whole process advances normally. It was evidenced that intermediate thermal treatment and degassing steps were necessary to improve the attachment of the different functional groups onto the iron oxide NPs. It was found that the optimal decorating conditions had to do with the combination of carboxylic acid-modified initially ‘uncapped’ iron oxide NPs with amino-functionalized SiO_2_ dimples. It seems that the initial surface state of the iron oxide NPs was the dominant parameter that affected the decoration efficiency (with the NP DLS size playing a possible secondary role). We have also tested our best composite for the detection and removal of As(v) from spiked water. Although the performance of this material in arsenic removal was marginal in terms of rate of mass detected, the remediation process was environmentally benign and the fate of the material used was well controlled. This makes it promising as a supplementary low cost means for the detection of heavy metal pollutants together with more robust techniques. Further work on the optimization of the morphology and surface chemistry of the composite, aiming to render it more appealing for a variety of applications is ongoing. Still, so far mostly the decoration of one-patch or two-patch non-magnetic structures with magnetic NPs has been reported.^35^ Our present work shows that in principle the decoration of multiple-patch silica dimples is also feasible. As a next step, the variation of the dimples number and therefore the ability of the nanocomposites to be readily attracted in a faster way upon applying a magnetic gradient at the separation time will be pursued. Since we have shown that our strategy can be transferred from the decoration of gold NPs to the one of iron oxide NPs, it can be probably extended to a range of different NPs with varying composition. In this way, we shall be able to get advantage of the enhanced total surface area that such isolated decorated particles can offer.

## Experimental

### Materials

1,10-Phenanthroline monohydrate (97%) was purchased from Acros Organics, UK. Sodium hydroxide (50%) was bought from Alfa Aesar, UK. Ethanol (96%) was purchased from Haymankimia, UK. 3-Amino-propyltrimethoxysilane (APS) (97%), ethyl chloroformate (≥98%), hydrochloric acid (37%), hydroxylamine hydrochloride (98%), iron(ii) chloride tetrahydrate, *N*,*N*-dimethylformamide (DMF) (≥99.8%), sodium acetate, succinic anhydride, and triethylamine (TEA) (98%) were purchased from Sigma-Aldrich, UK. Acetone, methanol (≥99.8%), glycerol (99.5%) and nitric acid (68%) were purchased from VWR, UK.

The pre-synthesized citrate-capped and uncapped maghemite NPs were synthesized according to a protocol described previously based on a modified polyol route. The use of nitric acid as an oxidizing agent ensured that any magnetite phases would be oxidized to maghemite.^36^ The amine-functionalised silica dimples were produced as explained elsewhere.^24^ The precursor batch of polystyrene–silica multipods was obtained from 85 nm silica seeds. It was transformed into polystyrene/silica hexapods with a morphology purity of 83%. The main side-products are tetrapods (4%) and pentapods (13%).^37^ Before the polystyrene dissolution, the silica core was regrown up to 185 nm. This means that the dimple depth is 50 nm. A small amount of ‘impurities’, *i.e.* silica particles with only 4 or 5 dimples, may be spotted at the TEM grids upon extended survey.^35^

### Instruments

UV-Vis characterisation was used to determine the concentration of the pre-synthesized iron oxide NPs using a Spectramax M2/M2e UV/Vis/NIR spectrophotometer. The *ζ*-potential values and DLS size measurements of the samples were determined with a Malvern Zetasizer Nano-ZS. IR spectra were obtained from solid samples using a Fourier transform IR spectrometer (Perkin Elmer Spectrum 2000 with Autoimage). Particle size and morphology were observed by TEM. The images were acquired using a JEOL JEM 1200-EX microscope operating at an acceleration voltage of 120 kV. For TEM imaging, a drop of NP dispersions was deposited on carbon-coated Cu grids. The crystal structure of the NFs was studied with a PanAlytical X-ray diffractometer (X'Pert Pro) using Co Kα radiation (*λ* = 1.789010 Å). Diffraction patterns were recorded from 2*θ* = 20° to 100°. Specimens were prepared by placing dry powders of the NFs on a zero-background Si wafer, which has no background noise at the measurement range. Magnetic measurements were performed with a Quantum Design hybrid Superconducting Quantum Interference Device-Vibrating Sample Magnetometer (SQUID-VSM). TGA was carried out using a Discovery TGA (TA instruments) under a N_2_ atmosphere between room temperature up to over 500 °C with a heating rate of 10 °C min^−1^.

### Iron oxide nanoparticles quantification

The pre-synthesized iron oxide NPs capped with citrate and the pre-synthesized uncapped iron oxide NPs were characterised through UV-Vis spectroscopy to identify the concentration of the samples. First of all, 2 mg mL^−1^ of iron(ii) chloride tetrahydrate (a standard sample) was diluted to 0.2 mg mL^−1^, 0.1 mg mL^−1^, 0.05 mg mL^−1^, 0.025 mg mL^−1^, 0.0125 mg mL^−1^ and 0.00625 mg mL^−1^ using deionized water. At this step, sodium acetate, hydroxylamine hydrochloride, the prepared diluted sample and 1-10-phenanthroline monohydrate were mixed together. These procedures were repeated for both uncapped and capped types of samples. Once all the samples were prepared, they underwent UV-Vis characterisation where the standard sample results were used to construct the calibration curve and obtain concentration values.

### Iron oxide nanoparticles characterisation

The pre-synthesized iron oxide NPs were characterised through FTIR and DLS (*ζ*-potential measurements) in order to confirm the presence and the attachment of the functional groups on the surface of the iron oxide NPs. TEM characterisation was used to determine the size, shape and decoration of the iron oxide NPs onto the silica dimples.

Regarding the FTIR characterisation, the pre-synthesized citrate-capped iron oxide NPs were centrifuged and underwent a freeze-dry process to remove the remaining liquid and form a solid powder. The obtained solid powder was characterised through FTIR to confirm the presence of the functional group on the iron oxide NPs. For the *ζ*-potential values, the samples to be measured were prepared by adjusting the pH level in the range from 3 to 11, using diluted nitric acid and diluted sodium hydroxide solution in order to identify the isoelectric point of the pre-synthesized iron oxide NPs. The isoelectric point of the samples was determined due to the fact that different functional groups have been shown to behave in a distinct way in terms of their protonation and deprotonation behaviour and provide discrete isoelectric point.^18^ Thus, the changes in isoelectric point can be used to confirm the changes in the surface chemistry of the iron oxide NPs after the modification.

### Iron oxide nanoparticles stability test

0.1 M nitric acid was added into the pre-synthesized iron oxide NPs drop by drop to observe the changes in the physical properties of the sample. The observation includes precipitation/colloidal stability and the changes in the colour of the samples.

### Iron oxide nanoparticles surface silanation for amino group modification with APS

In 5 mL of pre-synthesized citrate-capped iron oxide NPs with a pH of 2.5 (adjusted using diluted nitric acid), 0.44 mL APS and 5 mL methanol were added while stirring at 900 rpm. The mixture was left stirring overnight at room temperature. Next, the sample was placed in an oil bath to remove methanol and water at 40 °C and 80 °C under vacuum for 1 h at each temperature. Later, further dehydration process was carried out under vacuum conditions at 110 °C for 2 h to remove the remaining water. After the heat treatment, the dehydrated sample was left to cool down naturally to room temperature. Once methanol and water were successfully removed, the sample was washed with water and acetone at a ratio of 3 : 7, three times, by centrifugation at 7800 rpm for 20 min. Finally, the pH level of the sample was adjusted down again to pH 3 using diluted nitric acid while stirring at room temperature. The changes in pH level to an acidic condition initiate the peptisation within the mixture to obtain APS-modified iron oxide NPs.^19^ This protocol was repeated in the case of the pre-synthesized uncapped iron oxide NPs.

### APS-modified iron oxide nanoparticles characterisations

Once the pre-synthesized iron oxide NPs were successfully modified by APS in order to attach the amino group onto the surface, TEM, FTIR and DLS (*ζ*-potential measurements) characterisations were used to confirm the surface modification, size, shape and distribution of the obtained APS modified-iron oxide NPs. In order to monitor the changes in the surface functionality of the iron oxide NPs, FTIR characterisation was used. In addition, the *ζ*-potential values of the APS-modified iron oxide NPs with varying pH were recorded.

### APS-modified iron oxide nanoparticles modification with carboxylic acid groups

The APS-modified iron oxide NPs obtained from the previously described silanation process were transferred from water to DMF by centrifuging at 7800 rpm for 20 min twice. The sample was re-dispersed in DMF using a vortex mixer and sonication with an addition of 164 μL TEA. Then the mixture was centrifuged at 7800 rpm for 20 min. After the centrifugation, the sample was transferred to a round bottom flask for dehydration under vacuum for 2 h at 60 °C in an oil bath. Next, 118 mg of succinic anhydride was added into the sample and allowed to react overnight at 60 °C under a blanket of inert gas (N_2_). The sample was collected and washed twice with ethanol at 8300 rpm for 30 min and DMF at 7800 rpm for 20 min, respectively. Next, 164 μL of TEA was added followed by another centrifugation. The sample was then re-dispersed again in DMF and dehydrated in the oil bath for the next 2 h at 60 °C. After the dehydration process, the sample was left stirring overnight at room temperature. As a result, the amino group on the surface of the iron oxide NPs was converted to form the carboxylic acid-modified iron oxide NPs. FTIR and *ζ*-potential measurements were carried out also in this stage to determine the functional groups present and the isoelectric point.

### Carboxylic acid-modified iron oxide nanoparticles and amine-functionalised silica dimple grafting

For the preparation of the amine-functionalised silica dimples, 164 μL of TEA was added to 6 mL of pre-synthesized silica dimples dispersed in DMF (containing approximately 6 × 10^14^ dimples per L) and centrifuged at 7800 rpm for 20 min. This was followed by a dehydration process under vacuum for 1 h at 50 °C.

Alongside with that, the obtained carboxylic acid-modified iron oxide NPs dispersed in DMF, as shown previously herein, were centrifuged and re-dispersed in DMF at 7800 rpm for 20 min. Next, 26.24 μL of TEA and 8.95 μL of ethyl chloroformate were added into this solution and mixed using the vortex device and a roller mixer until the solution was completely homogenised to initiate the reaction. After the mixing, the dehydrated amine-functionalised silica dimples were added to the above solution and left to homogenise and react on the roller mixer overnight at a constant speed (room temperature). This solution was then transferred into 10 mL water and washed with 10 mL water and 0.1 mL of TEA at 650 rpm for 20 min three times, in order to remove excess iron oxide NPs. Lastly, the obtained iron oxide NPs-grafted silica dimples were characterised by TEM to observe the decoration of the silica dimples.

### Arsenic removal from water

To investigate the arsenic adsorption performance of the iron oxide-decorated silica dimples, the particles were submerged in 20 mL of As(v) solution. In particular, a solution of Na_2_HAsO_4_·7H_2_O in aqueous medium was prepared. The contact time was approximately 1 h, the concentration used was 10 mg L^−1^, and diluted hydrochloric acid was added to adjust the pH value to 6. X-ray photoelectron spectroscopy (XPS) spectra were obtained with an Axis Ultra DLD system by KRATOS in ultrahigh-vacuum conditions using a monochromated Al-Kα1 X-ray beam as the excitation source. The pass energy was 160 eV for survey scans and 40 eV for high resolution spectra. The spectra were calibrated in terms of charging-induced shifts by considering the C1s peak (originating from carbon surface contamination) to be located at 284.6 eV.

## Conflicts of interest

There are no conflicts to declare.

## Supplementary Material

RA-011-D0RA09907D-s001
