# Supplementary material for: Dimpled SiO2@γ-Fe2O3 nanocomposites – fabrication and use for arsenic adsorption in aqueous medium
Source: RSC Adv. 2021 Jan 5;11(3):1343–53. doi: 10.1039/d0ra09907d (PMC8693525; doi:10.1039/d0ra09907d)
Supplement: RA-011-D0RA09907D-s001 [file RA-011-D0RA09907D-s001.pdf]

Electronic Supplementary Material (ESI) for RSC Advances.
This journal is © The Royal Society of Chemistry 2020

Electronic Supplementary Information

for

Dimpled SiO_2@γ-Fe_2O_3 nanocomposites – fabrication and use for arsenic adsorption in aqueous medium

Saruta Deeprasert,[a,b] Lilin Wang,[a,b] Konstantinos Simeonidis,[c] Nguyen Thi Kim Thanh,[a,b,*] Etienne Duguet,[d,*] Stefanos Mourdikoudis,[a,b,*]

[a] Biophysics Group, Department of Physics and Astronomy, University College London, London, WC1E 6BT, UK

[b] UCL Healthcare Biomagnetic and Nanomaterials Laboratories, 21 Albemarle Street, London W1S 4BS, UK

[c] Department of Physics, Aristotle University of Thessaloniki, 54124 Thessaloniki, Greece

[d] Univ. Bordeaux, CNRS, Bordeaux INP, ICMCB, UMR 5026, F-33600 Pessac, France

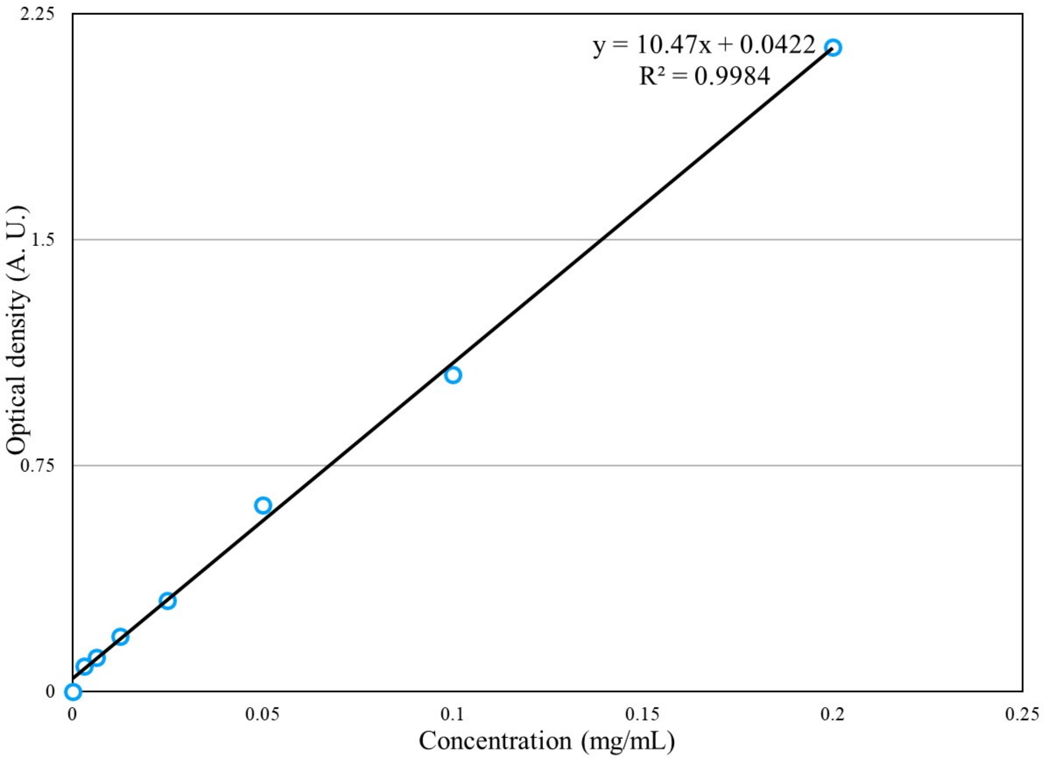

Fig. S1 Calibration curve created by UV-Vis measurements of Fe-containing samples with known concentration.

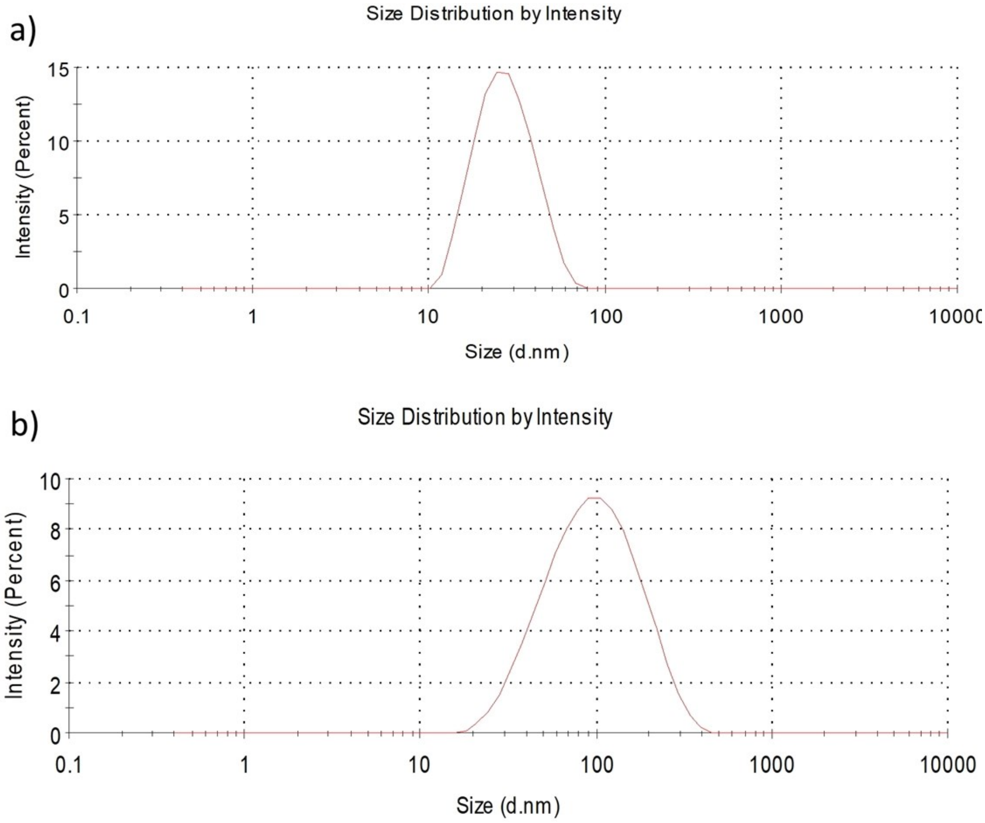

Fig. S2 DLS measurements for citrate-capped (a) and uncapped γ-Fe_2O_3 NFs (b).

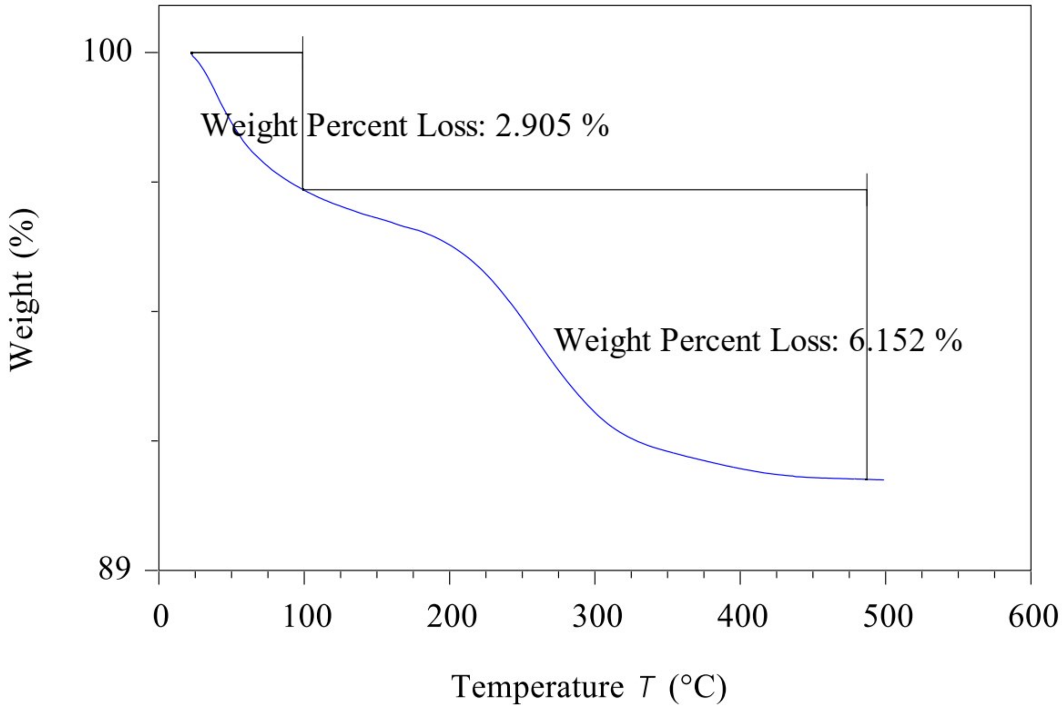

Fig. S3 Thermogravimetric analysis of maghemite NFs.

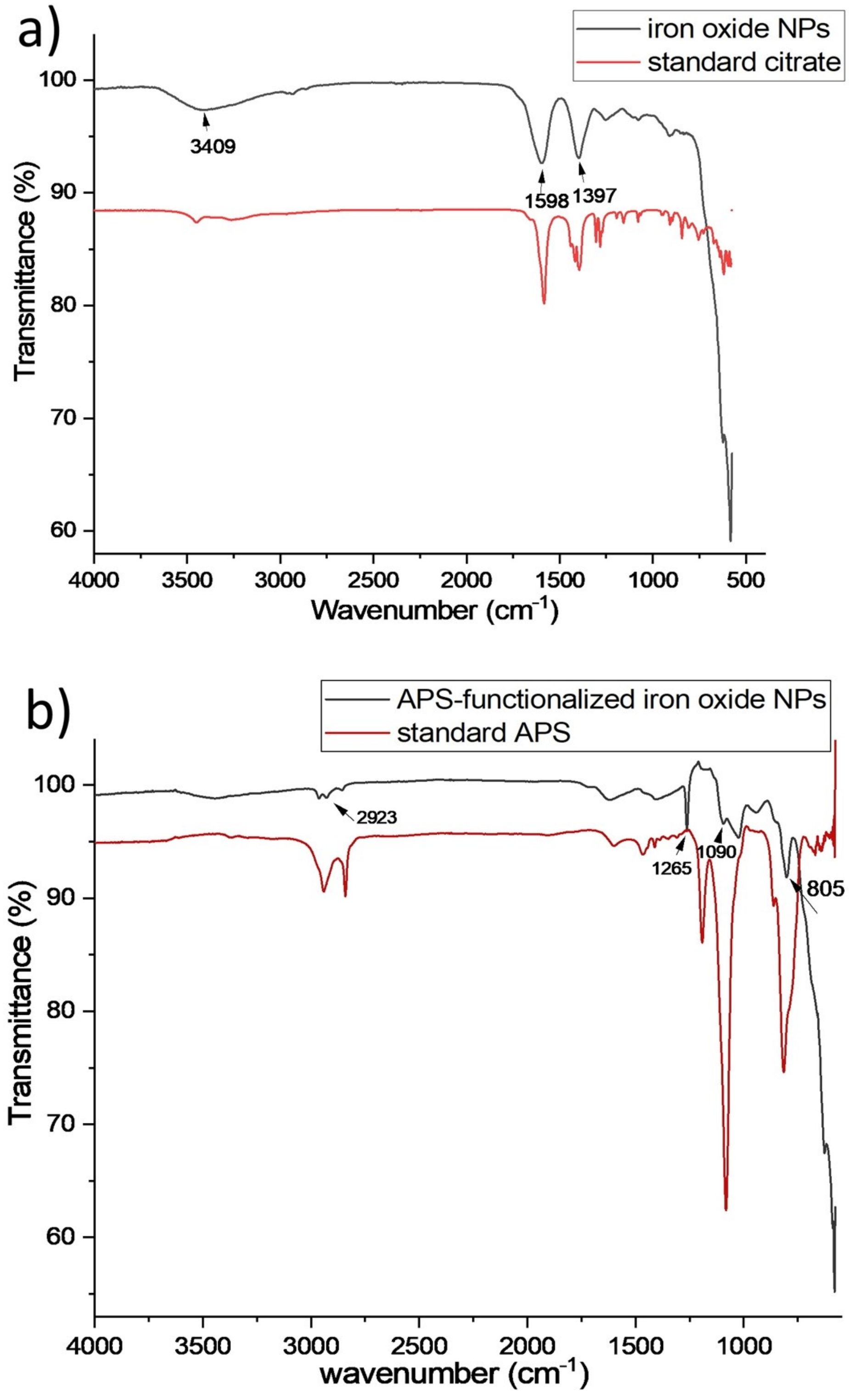

Fig. S4 FTIR spectra of: a standard citrate sample and of the pre-synthesized iron oxide NPs (a), APS molecule and APS-modified iron oxide NPs (b).

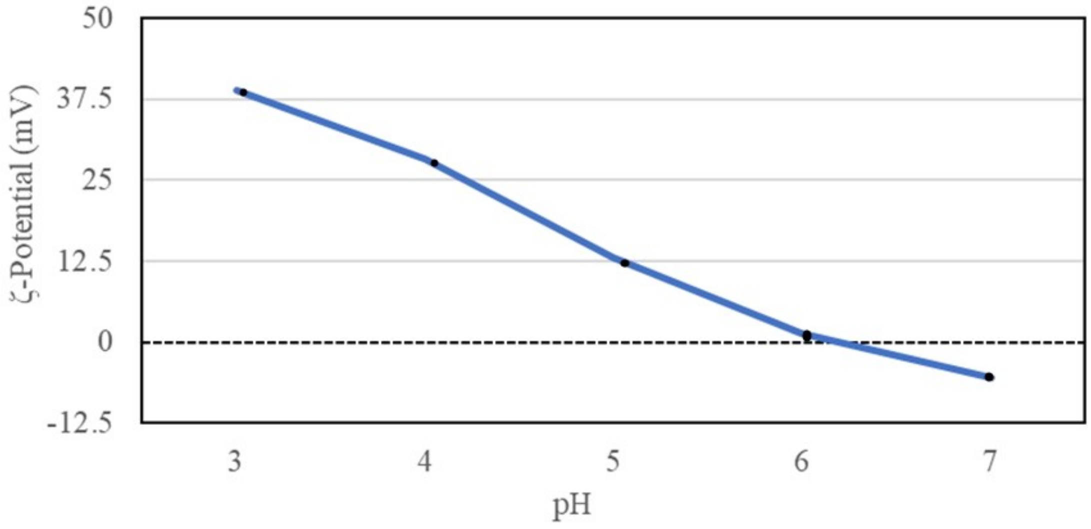

Fig. S5 ζ-potential measurements of the initial iron oxide NPs in a range of different pH values.

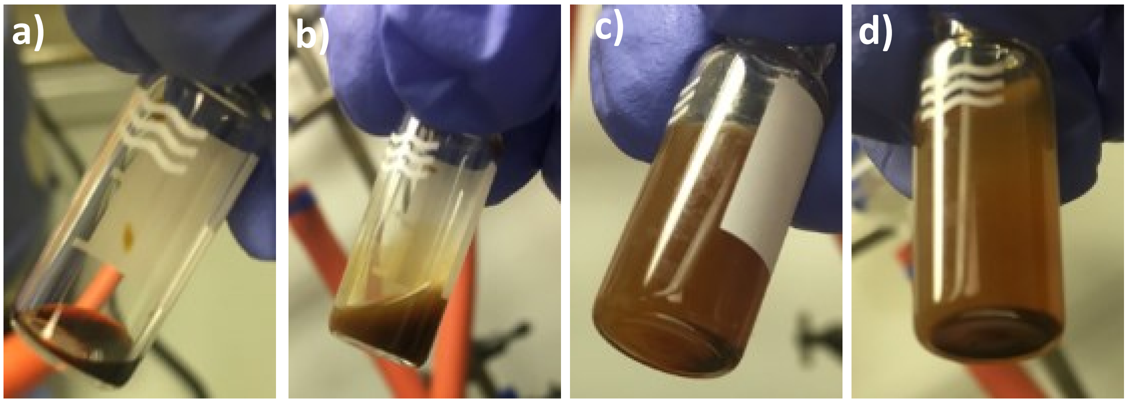

Fig. S6 Photographs of the dispersion of pre-treatment iron oxide NPs: a) no nitric acid added, b) 0.5 ml of 0.1 M nitric acid added, c) 1 ml nitric of 0.1 M nitric acid and d) 1.5 ml of 0.1 M nitric acid added.

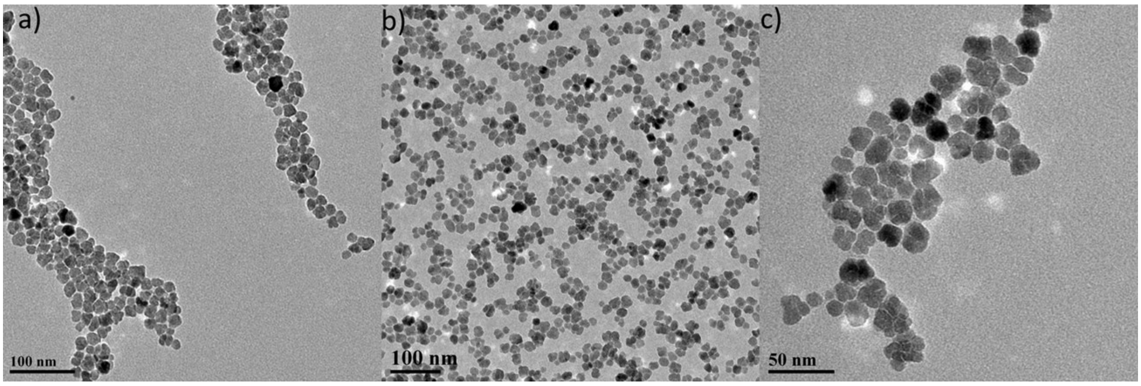

Fig. S7 TEM images of APS-modified iron oxide nanoparticles

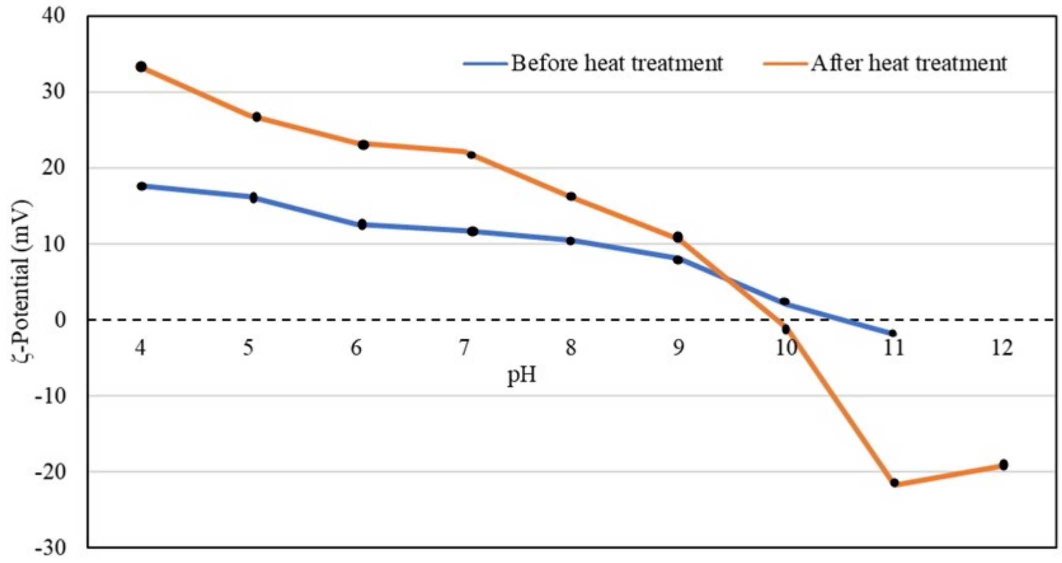

Fig. S8 ζ-potential measurement of APS-modified iron oxide NPs before and after heat treatment at 110 °C

Table S1: FTIR absorption frequencies of standard APS[S1]

Wavenumber (cm^{-1})	Intensity	Assignments
816	Strong	Si-O-CH_3 stretching
1083	Very Strong	Si-O-C stretching
1118	Strong	O-CH_3 rocking
1455	Medium	CH_2 shearing
1588	Weak	NH shearing
2842	Strong	O-CH_3 stretching
2930	Strong	CH_2 stretching

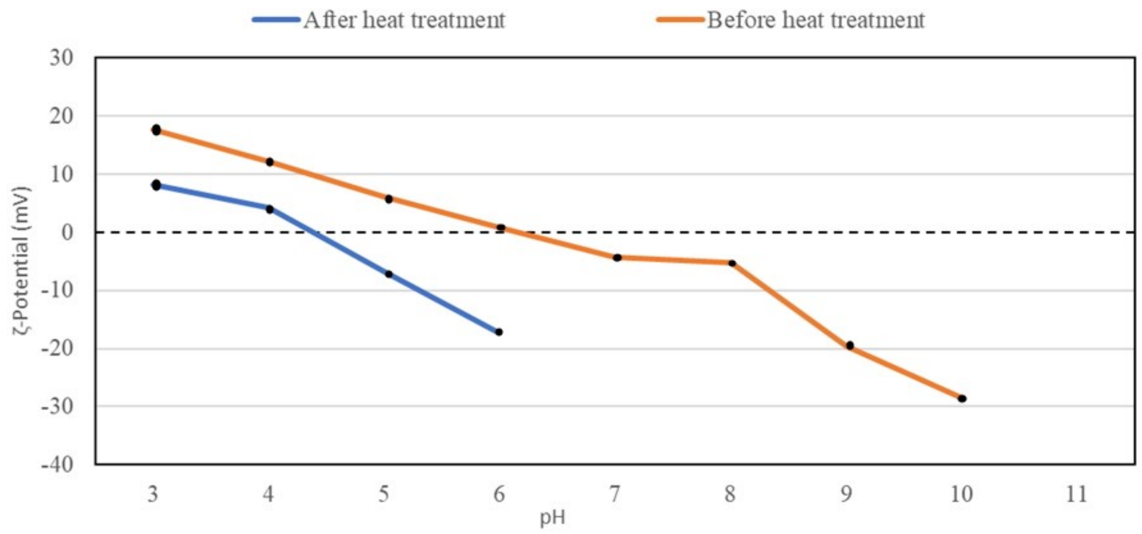

Fig. S9 ζ-potential measurements of the carboxylic acid-modified iron oxide nanoparticles before and after heat treatment.

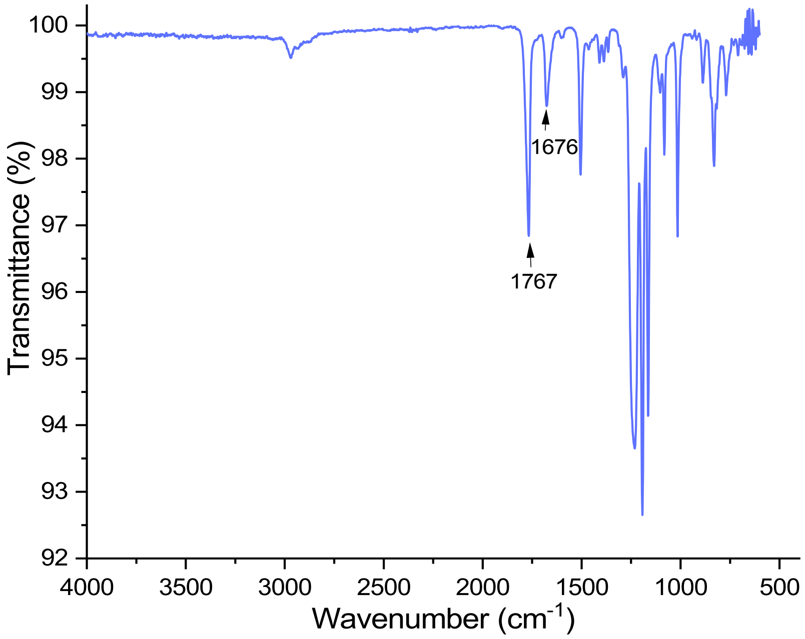

Fig. S10 FTIR spectrum of carboxylic acid-modified iron oxide nanoparticles

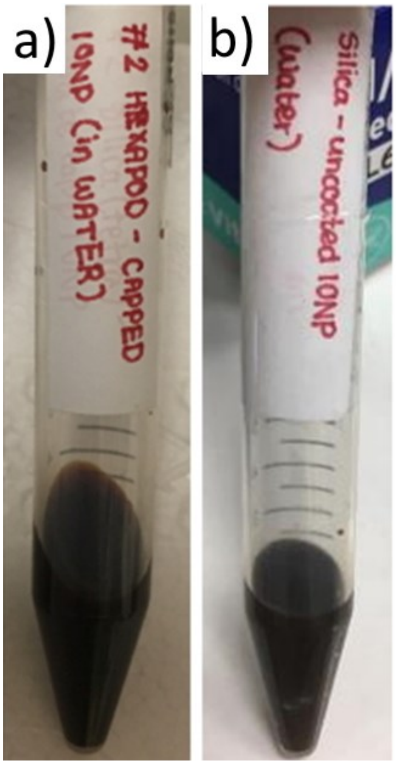

Fig. S11 Visual images of the colloidal dispersion of nanocomposites consisting of silica dimples and citrate-capped iron oxide NFs a) and uncapped iron oxide NFs (b). The latter sample was used for As removal

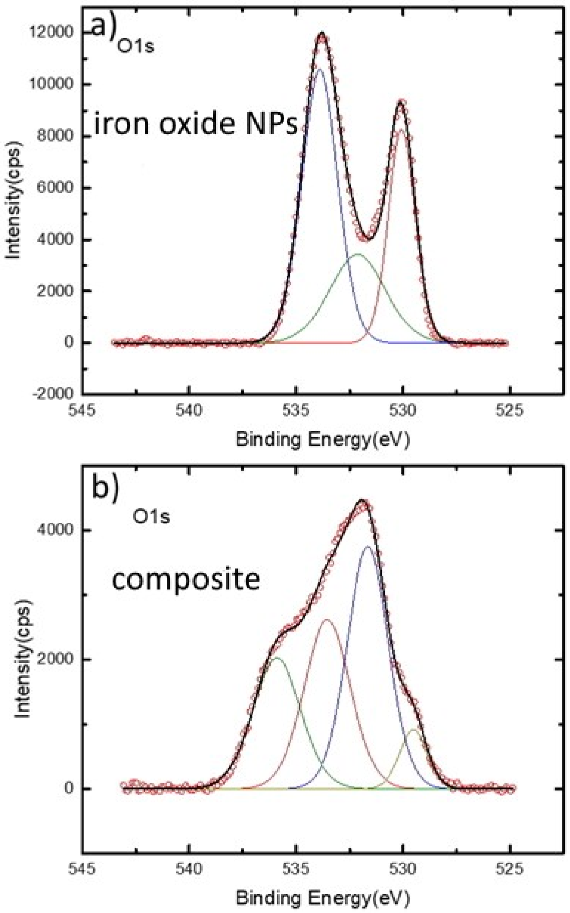

Fig. S12 High-resolution XPS spectra at the O1s: a) iron oxide NPs, b) silica dimples@iron oxide NPs

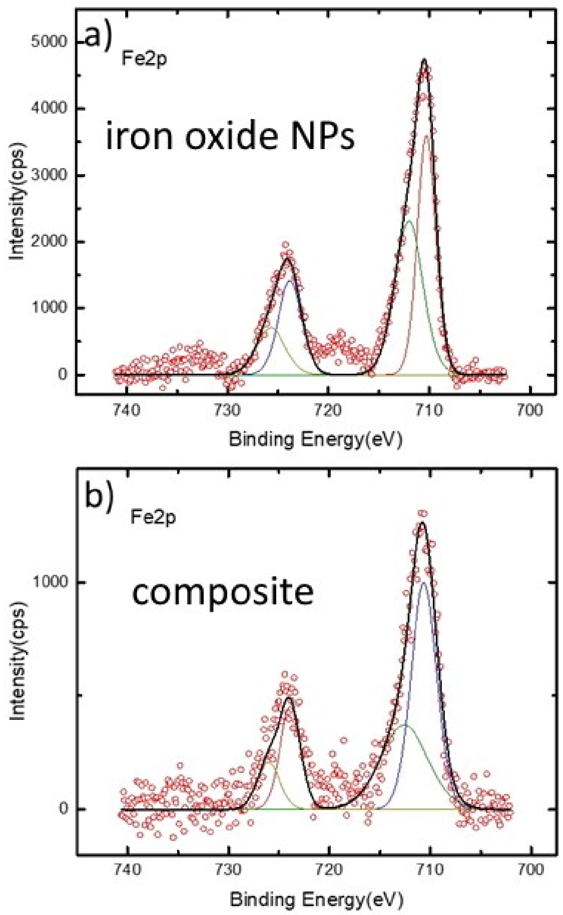

Fig. S13 High-resolution XPS spectra at the Fe2p a) iron oxide NPs, b) SiO_2 dimples@iron oxide NPs

Table S2: As-to-Fe mass ratio determined by XPS for various As(V) concentrations and acidities for silica dimples@iron oxide NPs

pH	As(V) concentration (mg/L)	As/Fe
6	1	0.011
6	5	0.028
6	10	0.052
6	20	0.097
8	10	0.033
10	10	0.024

(S1) S. Mornet, J. Portier and E. Duguet, *J. Magn. Magn. Mater.* 2005, **293**, 127
References
